# Comparing bioinformatic pipelines for microbial 16S rRNA amplicon sequencing

**DOI:** 10.1371/journal.pone.0227434

**Published:** 2020-01-16

**Authors:** Andrei Prodan, Valentina Tremaroli, Harald Brolin, Aeilko H. Zwinderman, Max Nieuwdorp, Evgeni Levin

**Affiliations:** 1 Department of Experimental Vascular Medicine, Amsterdam University Medical Centers, Amsterdam, The Netherlands; 2 Wallenberg Laboratory for Cardiovascular and Metabolic Research, Department of Molecular and Clinical Medicine, Institute of Medicine, Sahlgrenska Academy, University of Gothenburg, Gothenburg, Sweden; 3 Department of Clinical Epidemiology, Biostatistics and Bioinformatics, Amsterdam University Medical Centers, Amsterdam, The Netherlands; 4 Horaizon BV, Delft, the Netherlands; Seoul National University College of Medicine, REPUBLIC OF KOREA

## Abstract

Microbial amplicon sequencing studies are an important tool in biological and biomedical research. Widespread 16S rRNA gene microbial surveys have shed light on the structure of many ecosystems inhabited by bacteria, including the human body. However, specialized software and algorithms are needed to convert raw sequencing data into biologically meaningful information (i.e. tables of bacterial counts). While different bioinformatic pipelines are available in a rapidly changing and improving field, users are often unaware of limitations and biases associated with individual pipelines and there is a lack of agreement regarding best practices. Here, we compared six bioinformatic pipelines for the analysis of amplicon sequence data: three OTU-level flows (QIIME-uclust, MOTHUR, and USEARCH-UPARSE) and three ASV-level (DADA2, Qiime2-Deblur, and USEARCH-UNOISE3). We tested workflows with different quality control options, clustering algorithms, and cutoff parameters on a mock community as well as on a large (N = 2170) recently published fecal sample dataset from the multi-ethnic HELIUS study. We assessed the sensitivity, specificity, and degree of consensus of the different outputs. DADA2 offered the best sensitivity, at the expense of decreased specificity compared to USEARCH-UNOISE3 and Qiime2-Deblur. USEARCH-UNOISE3 showed the best balance between resolution and specificity. OTU-level USEARCH-UPARSE and MOTHUR performed well, but with lower specificity than ASV-level pipelines. QIIME-uclust produced large number of spurious OTUs as well as inflated alpha-diversity measures and should be avoided in future studies. This study provides guidance for researchers using amplicon sequencing to gain biological insights.

## Introduction

Microbial surveys based on 16S rRNA gene amplicon sequencing are an important tool in environmental and biomedical research [1–2]. Microbial community structure can provide valuable insights not only into the workings of natural ecosystems, but increasingly into the relationship between the human host and its bacterial colonizers. Rapid progress in DNA sequencing technology has provided ever-increasing outputs coupled with lowered costs, facilitating an explosion in amplicon sequencing studies [3]. Unfortunately, these studies are vulnerable to potential biases introduced along the workflow and there is a lack of consensus regarding best practices [4–5]. This paper aims to provide researchers (e.g. ecologists, microbiologists, biomedical researchers) with a overview of the strengths and weaknesses of six of the most popular current bioinformatic pipelines for 16S rRNA gene amplicon sequencing. While this selection is not a comprehensive set, it includes some of the most used (QIIME [6], MOTHUR [4], and USEARCH [5]) as well as more recent options (DADA2 [6] and Qiime2-Deblur [7–8]). Three of these pipelines cluster sequences at (typically) 97% identity into Operational Taxonomical Units (OTUs): QIIME-uclust, MOTHUR and USEARCH-UPARSE. The other three (Qiime2-Deblur, DADA2, and USEARCH-UNOISE3) attempt to reconstruct the exact biological sequences present in the sample, so-called Amplicon Sequence Variants (ASVs) [9]. ASVs are referred to by other authors as “zero noise OTUs” [10] or “sub-OTUs” [7].

The pipelines benchmarked here may perform better than reported in this paper if their parameters are customly tuned for an individual dataset. However, we believe that the vast majority of users employ either default or author-recommended settings. We therefore aimed to compare pipelines under these typical conditions in order to match the most plausible use scenarios. We examined the effect of different quality filtering steps (for QIIME-uclust, Qiime2-Deblur, and DADA2) and of different clustering algorithms and cutoffs (for MOTHUR), as we deemed these to be the most likely pipeline variations users might attempt. While other benchmarking studies have been published, they relied only on simulated (synthetic) reads [11] or on very small data sets [12].

In this paper, pipelines were compared using a mock sample sequenced repeatedly over multiple sequencing runs as well as a large (N = 2170 individuals) fecal sample dataset from the “Healthy Life in an Urban Setting” (HELIUS) multi-ethnic study [13–14]. We examined the specificity and sensitivity of each workflow (e.g. number of spurious OTUs/ASVs produced), the quantitative agreement between the inferred relative abundances, as well as any pipeline-specific effects on downstream alpha-diversity measures.

## Material and methods

### Datasets

#### Mock community

Genomic DNA from the Microbial Mock Community B (Even, Low concentration), v5.1L (Catalog no. HM-782D, obtained through BEI Resources, NIAID, NIH as part of the Human Microbiome Project) was sequenced in three separate runs. Details of mock composition are included in S1 Table. The mock contains DNA from 20 bacterial strains in equimolar (Even) ribosomal RNA operon counts (100000 copies per organism per μL). Two of the strains (*Bacteriodes vulgatus* and *Clostridium beijerinckii*) have multiple sequence variants in the V4 region of the 16S rRNA gene. *B*. *vulgatus* has three variants (in a 5:1:1 ratio), whereas *C*. *beijerinckii* has two variants (in a 13:1 ratio). The 16S rRNA sequences of *Staphylococcus aureus* and *Staphylococcus epidermidis* are identical in the V4 region. Therefore, the mock contains a total of 22 variants (ASVs) of the 16S gene in the V4 region. These sequences correspond to 19 OTUs when clustered at 97% identity. The mock community was sequenced three times in different sequencing runs. The mock raw sequence data is publicly available (https://github.com/andreiprodan/mock-sequences).

#### HELIUS fecal samples dataset

A total of 2170 fecal samples obtained from adult individuals from six ethnic groups in Amsterdam, the Netherlands (the HELIUS study) were sequenced. Cohort information and detailed sample collection and processing protocols have been previously described [13–14]. The HELIUS fecal sample dataset contained 177.08 million paired-end reads obtained from 17 individual sequencing runs. All raw sequencing data from this dataset is available on the European Genome-phenome Archive repository (accession no. EGAD00001004106).

### Library preparation and sequencing

Library preparation and sequencing was performed at the Wallenberg Laboratory (Sahlgrenska University of Gothenburg, Sweden). Total genomic DNA was extracted from a 150 mg fecal sample aliquot using a repeated bead beating method as previously described [15]. Fecal microbiome composition was profiled by sequencing the V4 region of the 16S rRNA gene on an Illumina MiSeq instrument (Illumina RTA v1.17.28; MCS v2.5) with 515F and 806R primers designed for dual indexing [16] and the V2 Illumina kit (2x250 bp paired-end reads). 16S rRNA genes from each sample were amplified in duplicate reactions in volumes of 25 μL containing 1x Five Prime Hot Master Mix (5 PRIME GmbH), 200 nM of each primer, 0.4 mg/ml BSA, 5% DMSO and 20 ng of genomic DNA. PCR was carried out under the following conditions: initial denaturation for 47 min at 94°C, followed by 25 cycles of denaturation for 45 sec at 94°C, annealing for 60 sec at 52°C and elongation for 90 sec at 72°C, and a final elongation step for 10 min at 72°C. Duplicates were combined, purified with the NucleoSpin Gel and PCR Clean-up kit (Macherey-Nagel) and quantified using the Quant-iT PicoGreen dsDNA kit (Invitrogen). Purified PCR products were diluted to 10 ng/μL and pooled in equal amounts. The pooled amplicons were purified again using Ampure magnetic purification beads (Agencourt) to remove short amplification products. Libraries for sequencing were prepared by mixing the pooled amplicons with PhiX control DNA purchased from Illumina. The input DNA had a concentration of 3 pM and contained 15% PhiX and resulted in the generation of about 700K clusters/mm^2^ and an overall percentage of bases with quality score higher than 30 (Q30) higher than 70%.

### Pipelines and parameters

Six different pipelines were included in this comparison: QIIME (v.1.9.1) [17], MOTHUR (v.1.39.5) [4], DADA2 (1.7.0) [6], Qiime2 (v.2017.6.0)-Deblur [7,8], USEARCH (v.10.0.240)-UPARSE [18], and USEARCH (v.10.0.240)-UNOISE3 [10].

#### Paired-end reads merging and quality filtering

**Fig 1:**
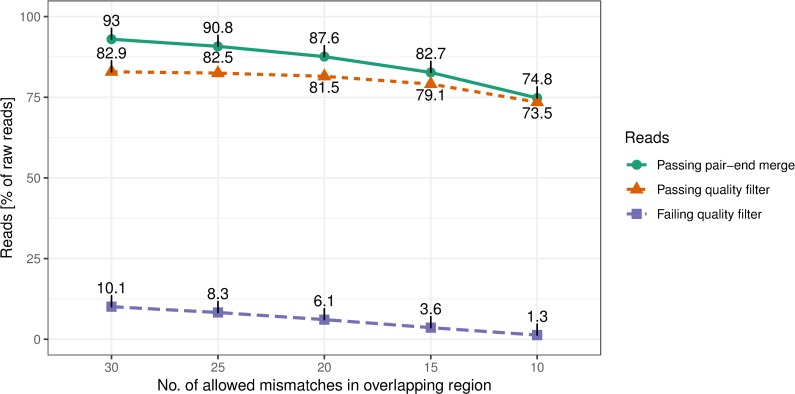
Effect of different USEARCH paired-end read merging parameters (“maxdiffs”).

In MOTHUR, the merging and quality filtering of reads (“screening”) is an integral part of the pipeline, not easily performed outside MOTHUR. We therefore used MOTHUR only with its internal read merging and filtering. In DADA2—in contrast to all other pipelines—denoising is always implemented separately on the forward and the reverse reads, with resulting ASVs merged at the end of the workflow. External merging / filtering is therefore not applicable to DADA2, while it can be integrated with relative ease into QIIME and Qiime2 workflows. We therefore implemented one workflow in QIIME-uclust and one workflow in Qiime2-Deblur where reads were merged and filtered externally using USEARCH. The author of USEARCH explicitly advises against using the default USEARCH read merging parameters for reads with a long overlap (e.g. MiSeq 2 x 250bp V4) and argues in favor of maximizing the proportion of reads that survive the merging step. We therefore benchmarked several settings for the merging step. Based on the outcomes (Fig 1), we chose 30 max. allowed differences in the overlapping region (“maxdiffs”) for the merging step (using the “fastq_mergepairs” command) and max. 1 expected errors (“fastq_maxee”) as a quality filter threshold (using the “fastq_filter” command). The rationale was to use permissive parameters in the merging step in order to fully exploit the error correction made possible by the overlapping of the reads (e.g. a lower Q-score sequencing error in the reverse read can be rectified using the higher Q-score correct base call from the forward read). Strict thresholds (i.e. low “maxdiffs”) discard read pairs where mismatches due to an error on one read might have been easily corrected using the complementary read. As Fig 1 shows, more relaxed merging parameters resulted in around 10% more of total raw reads (82.9% compared to 73.5%) passing the quality filter. These merging / filtering parameters were used in the USEARCH-UPARSE, USEARCH-UNOISE3, QIIME-uclust (e30.ee1), and Qiime2-Deblur (e30.ee1) flows. Expected error-based read quality filtering is described in detail in Edgar et al. 2015 [19].

#### QIIME-uclust

In the typical QIIME-uclust workflow, forward and reverse reads are merged using the “multiple_join_paired_ends.py” script. Subsequently, quality control and demultiplexing are performed simultaneously using the “multiple_split_libraries_fastq.py” script, which truncates the reads if more than r (default 3) consecutive bases do not have a Q-score higher than q (default 3). Reads are discarded if, after trimming, the read length drops to less than p (default 0.75) of initial length. By default, no ambiguous bases (“N”) are allowed (default is 0). OTU clustering was performed using the “pick_open_reference_otus.py” script, with all default parameters. This script implements the latest QIIME open reference OTU clustering [20]. In brief, it performs closed reference clustering against the Greengenes (v.13.8) 97% OTU database, using UCLUST v.1.2.22q [5]; reads that do not map in this first step are subsampled (default proportion of subsampling = 0.001) and used as new centroids for a *de novo* OTU clustering step. Remaining unmapped reads are subsequently closed-reference clustered against these *de novo* OTUs. Finally, another step of *de novo* clustering is performed on the remaining unmapped reads.

Three different QIIME-uclust workflows were run using different merging and quality control parameters. One QIIME-uclust flow used all default parameters (“QIIME-uclust (default)”), while another was run with the q parameter set to 19 instead of default 3 (“QIIME-default (Q20)”). For the 3rd flow the read merging and quality control steps were performed outside QIIME, using USEARCH with the same parameters used in the USEARCH flows: max. 30 allowed mismatches in the overlapping region for merging, max. 1 allowed expected error per merged read for filtering (“QIIME-uclust (e30.ee1)”).

#### MOTHUR

In MOTHUR, paired-end reads are merged using the “make.contigs” command. This aligns the forward and reverse reads and, if a position in the overlapping region has different base calls in the forward read versus the reverse read, compares the forward read Q-score and the reverse read Q-score for that position. If one of the two Q-scores is at least “deltaq” points higher than the other (default deltaq is 6), then the merged read will use the respective base call. Otherwise, if the difference between the forward and the reverse base call Q-scores is less than deltaq, the base at that position is re-labelled as ambiguous (“N”}). Quality filtering is implemented with the”screen.seqs” command, which (by default) removes all merged reads containing ambiguous bases. Sequences are then deduplicated, aligned to a database (SILVA v.128), and pre-clustered (i.e. sequences with less than 2 base differences (“diffs”) from a more abundant sequence are merged with the more abundant sequence). Chimeric reads are removed (“chimera.vsearch”) and remaining uniques are clustered into OTUs.

Two different clustering algorithms were used in MOTHUR: Opticlust (the current default) and DGC (Distance-based Greedy Clustering).

Opticlust [21] is a distance-based algorithm and therefore requires a distance matrix to be constructed between all unique sequences. The size of this matrix scales with the square of the number of sequences and can thus become problematic with large datasets (since the matrix must fit into available RAM memory). This issue can be side-stepped with the “split.abund” command, removing sequences with extremely low abundance after the pre-clustering step. In this study, a cutoff of 3 was used for the MOTHUR-Opticlust flow, keeping only sequences with more than 3 counts in the entire dataset (“MOTHUR (Opticlust.3)”).

DGC uses Vsearch [22] (an open source alternative to USEARCH) in order to perform greedy clustering which does not require a distance matrix. Three different MOTHUR pipelines were run with DGC clustering, applying different cutoffs to the “split.abund” command after the preclustering step: “MOTHUR (DGC.0)” (cutoff 0, i.e. not removing any sequences), “MOTHUR (DGC.1)” (cutoff 1, i.e. only removing singletons, sequences that appear only once in the entire dataset), and “MOTHUR (DGC.3)” (cutoff 3, i.e. removing unique sequences with at most 3 counts in the entire dataset).

#### DADA2

DADA2 (Divisive Amplicon Denoising Algorithm 2) uses a parametric model to infer true biological sequences from reads. The model relies on input read abundances (true reads are likely to be more abundant) and distances (less abundant reads only a few base-differences away from a more abundant sequence are likely error-derived). Base Q-scores are used to calculate a substitution model, estimating a probability for each possible base substitution (e.g. A replacing G, G replacing T, etc). Based on this substitution model and on the input reads abundances and reciprocal distances, DADA2 uses a probability threshold to decide whether to assign counts from a less abundant, “error-derived” read to a more abundant, “true” sequence. DADA2 was run as an R script (in R v.3.4) using its R package (dada2 v.1.7).

In DADA2, reads are quality-filtered using the “filterAndTrim” function. Error rates are subsequently learned from a set of subsampled reads (i.e. 1 million random reads). Error rates are estimated separately for each sequencing run, since different runs may have different error profiles. Reads are then deduplicated and ASVs are inferred. Uniquely, DADA2 retains a summary of the quality scores associated with each unique sequence (the average of the positional qualities from deduplicated reads). These quality scores are subsequently used to perform ASV inference. Also, unlike all the other pipelines, DADA2 denoises the forward and the reverse reads independently. ASVs from the forward and reverse flows are only merged at the end of the workflow prior to the removal of chimeric ASVs, using “removeChimeraDenovo”.

Two DADA2 pipeline flows were run using different quality score filters. Both flows truncated reads by removing the last 10 bases from the forward reads and the last 40 bases from the reverse reads, as well as truncating / removing reads with ambiguous bases. The 1st DADA2 flow, “DADA2 (ee2)”, filtered (trimmed) reads to max. 2 allowed expected errors per read, while the 2nd, “DADA2 (no filter)”, did not use an expected error-based filter. Removing or truncating reads with ambiguous bases is mandatory in DADA2.

#### Qiime2-Deblur

Qiime2 [8] is the successor platform to QIIME. It incorporates several plugins, including DADA2 and Deblur. We used Qiime2 in combination with its Deblur plugin [7], following the flow for paired-end reads from the Qiime2 website (https://qiime2.org/). Raw reads were imported into a Qiime2 artifact before merging paired-end reads and quality filtering. An artifact is a Qiime2-specific file format which holds data as well as metadata, provenance, and version information. Reads were then denoised using the “deblur denoise-16S” command, trimming reads at a length of 250 bases.

Deblur compares sequence-to-sequence Hamming distances to an upper-bound error profile combined with a greedy algorithm [7]. Sequences are sorted by abundance, then the number of predicted error-derived reads is subtracted from the counts of neighboring reads based on their read-to-read Hamming distance. Any sequence whose abundance drops to 0 during this process is removed. The Deblur algorithm is applied to each sample independently.

Three different Qiime2-Deblur flows were run. In the first, “Qiime2-Deblur (default), merging and trimming were performed inside Qiime2 using the “vsearch” plugin [22] (for merging) and the “quality-filter q-score” plugin (for filtering) with default options”. Similar to the quality filtering in QIIME, the Qiime2 quality-filter truncates reads if more than 3 consecutive bases do not have a Q-score higher than 3 and discards them if post-trimming read length is less than 0.75 of initial length. No ambiguous bases are allowed. The second Qiime2 flow, “Qiime2-Deblur (Q20)”, used the same parameters as the first, but with the Q-score threshold (“p-min-quality”) set to 20 In the third Qiime2 flow, “Qiime2-Deblur (e30.ee1)”, read merging and quality control were performed outside Qiime2, using USEARCH with the same parameters used in the USEARCH flows.

#### USEARCH-UPARSE and USEARCH-UNOISE3

Both the UPARSE [18] and the UNOISE3 [10] pipelines are implemented in USEARCH [5]. The merging and filtering (covered in the “Paired-end reads merging and quality filtering” subsection) as well as deduplicating (“fastx_uniques” command) are therefore identical for UPARSE and UNOISE3 and only need to be performed once, before the pipelines branch off into OTU-level clustering (with UPARSE) or ASV-level denoising (with UNOISE3). Indeed, the author of USEARCH advises that UPARSE and UNOISE3 should be performed together (https://drive5.com/usearch/manual/faq_uparse_or_unoise.html).

UPARSE uses the UPARSE-REF greedy algorithm to infer errors using the concept of parsimony. In brief, UPARSE-REF aims to explain a given input sequence starting from sequences in a database, using the fewest possible number of events (i.e. PCR or sequencing errors). It constructs a model sequence using one or more sequences from the database (i.g. a single sequence representing a non-chimeric amplicon, or multiple concatenated segments representing a chimeric amplicon). Different penalty scores are given for chimeric crossover and for mismatches and the model sequence with the lowest total score is chosen as the true OTU sequence. Sequences are ranked in decreasing order of abundance, discarding singletons. Each input sequence is then compared to the current OTU set and to the maximum parsimony model sequence constructed using UPARSE-REF. If the model sequence is more than 97% identical to an existing OTU, the sequence is assigned to the respective OTU. It the model sequence is chimeric, the sequence is discarded. Finally, if the model is less than 97% identical to any existing OTU, the sequence is added to the existing OTU set. After all OTUs are found, all merged reads (including those dropped during quality filtering) are mapped against the OTUs to construct the OTU table.

The UNOISE3 flow ranks sequences in decreasing order of abundance, discarding sequences with less than 8 counts (the default min. abundance default threshold). The ASV set is initially empty. A model is then applied (Eq 1) to each input sequence in order to test whether its abundance (a_M_) is sufficiently large compared to the abundance of its closest sequence (a_C_) at Levenshtein distance *d*. The default value of the α parameter is 2. If Eq (1) holds, the input sequence becomes a new ASV; else, the input sequence it assigned to the nearest existing ASV. The final set of ASVs undergoes chimera filtering using the UCHIME2 [23] algorithm in *de novo* mode. Similar to UPARSE, the final step in the UNOISE3 flow is to map all merged reads to non-chimeric ASVs in order to construct the ASV table.

$$a_{M}/a_{C}\leq 1/2^{\alpha d+1}$$ (1)

Both the UPARSE clustering (the “cluster_otus” command) and the UNOISE3 denoising (the “unoise3” command) steps were executed with all default settings. In both flows, all samples in a data set were processed together, rather than individually, in order to achieve optimal sensitivity.

### Data analysis

All OTU/ASV tables produced by the pipelines were converted into phyloseq objects using the “phyloseq” package [24](v.1.24.2). Alpha diversity measures (richness, Chao1, Shannon index, inverse Simpson index) were calculated using the “estimate_richness” function from “phyloseq”. OTUs/ASVs were classified as “Exact” (perfect match to a true sequence in the mock community), “One-off” (at 1 Hamming distance away from a true sequence), or “Other” (at more than 1 Hamming distance from a true sequence). Sequence-to-sequence Hamming distances were calculated using the “stringdist” R package (v.0.9.5.1). “One-off” and “Other” were together labeled as “Spurious”.

Plots were constructed in R [25] (v.3.4) using the “ggplot2”[26] (v.3.1.0), “corrplot”[27] (v.0.84), and “VennDiagram”[28] (v.1.6.20) packages.

## Results and discussion

### Mock community

#### Sensitivity and specificity

**Fig 2:**
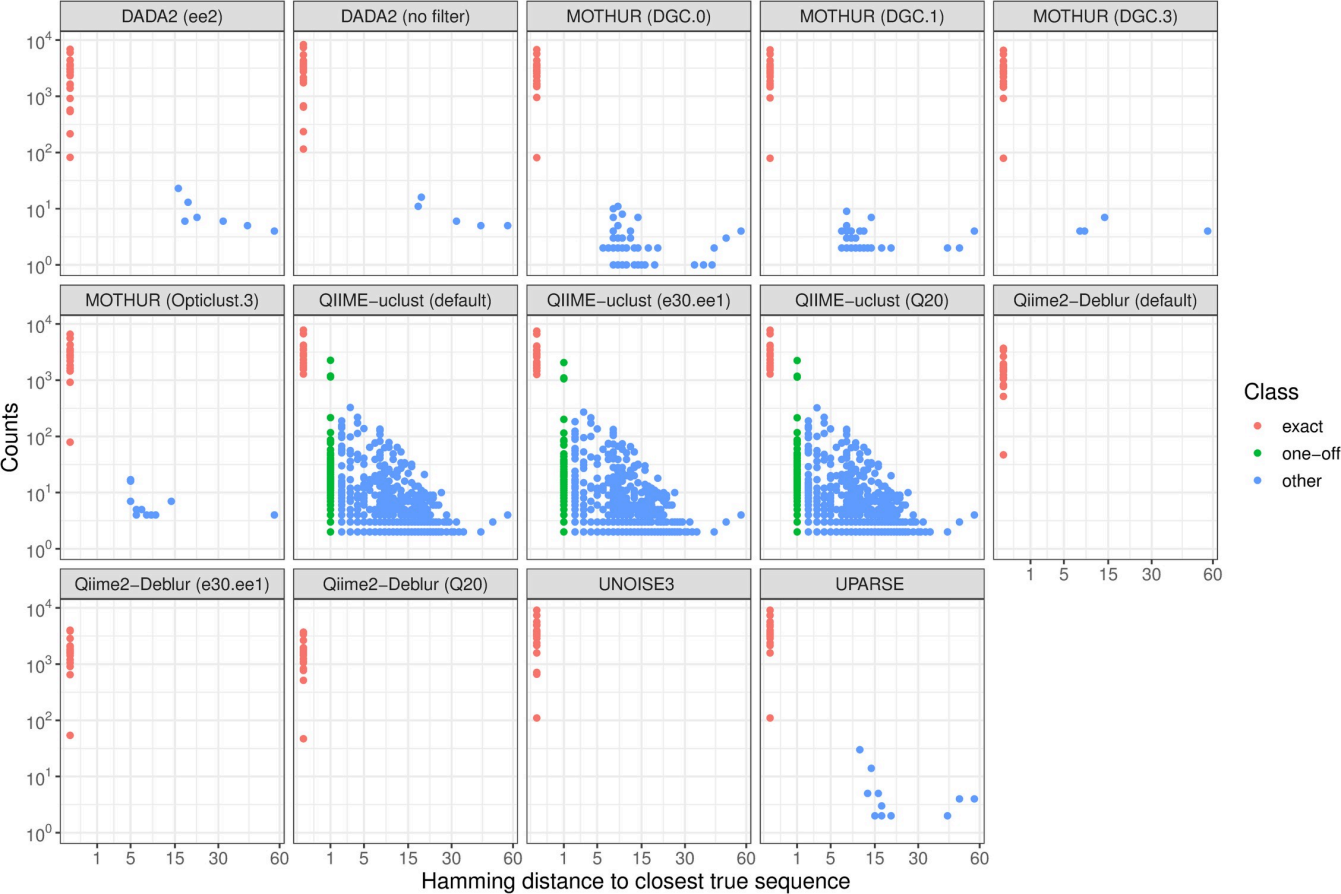
Hamming distance (no. of base differences) from each ASV/OTU sequence to the closest true sequence present in the mock community.

**Fig 3:**
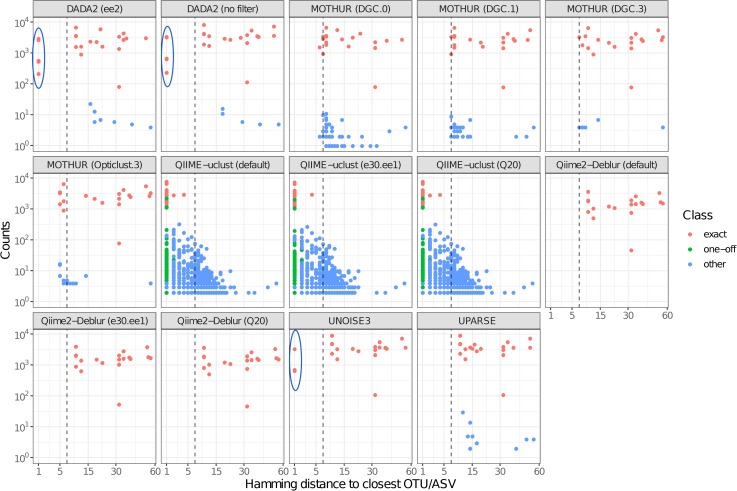
Hamming distance from each ASV/OTU sequence to the closest other ASV/OTU sequence. **Dashed line marks the Hamming distance = 7 threshold, corresponding to the 97% identity threshold for OTUs in V4 16S rRNA gene amplicons.** Blue ellipses highlight ASVs that are only 1 Hamming distance away from each other.

An overview of Exact, One-Off, and Spurious OTUs/ASVs produced by the different pipelines using reads from the three mock sequencing runs is shown in Table 1. The three mock sample runs had 36464, 84054, and 146653 paired-end reads, respectively. For each OTU/ASV sequence produced by the different flows, Hamming distances to the closest true sequence (Fig 2) and to the closest other OTU/ASV in the respective flow (Fig 3) were plotted as function of ASV/OTU abundance.

**Table 1:** Sensitivity and specificity over three mock sequencing runs. Values are reported as mean (standard deviation).

	Pipeline workflow	Exact	One-Off	Spurious
**OTU-level**				
**QIIME-uclust**	QIIME-uclust (default)	19 (0)^a^	134 (27)	412 (236)
	QIIME-uclust (e30.ee1)	19 (0)^a^	133 (31)	341 (198)
	QIIME-uclust (Q20)	19 (0)^a^	132 (26)	400 (232)
**MOTHUR**	MOTHUR (DGC.0)	19 (0)	none	48 (14)
	MOTHUR (DGC.1)	19 (0)	none	24 (8)
	MOTHUR (DGC.3)	19 (0)	none	5 (1)
	MOTHUR (Opticlust.3)	19 (0)	none	9 (4)
**UPARSE**	USEARCH-UPARSE	19 (0)	none	13 (7)
**ASV-level**				
**DADA2**	DADA2 (ee2)	21.7 (0.6)^b^	none	6 (4)
	DADA2 (no filter)	21.7 (0.6)^b^	none	5 (4)
**Qiime2-Deblur**	Qiime2-Deblur (default)	19 (0)	none	none
	Qiime2-Deblur (e30.ee1)	19 (0)	none	none
	Qiime2-Deblur (Q20)	19 (0)	none	none
**UNOISE3**	USEARCH-UNOISE3	21 (0)^c^	none	none

^a^ QIIME-uclust erroneously produced separate OTUs for the two *C*. *beijerinckii* sequence variants, even though they have only 1 bp difference. It did not detect *P*. *acnes* in one of the three mock runs.

^b^ DADA2 did not find the lower copy number *C*. *beijerinckii* variant in one of the three mock runs.

^c^ USEARCH-UNOISE3 could not differentiate the two *C*. *beijerinckii* variants (13:1 copy number ratio).

DADA2 showed the best sensitivity, detecting all 22 true ASVs present in the mock and was was the only pipeline able to differentiate sequences at single-base resolution even at high abundance ratios (e.g. the 13:1 ratio between the two *C*. *beijenrickii* variants). It only missed the low-abundance *C*. *beijenrickii* variant ASV in the sequencing run with the lowest number of raw reads. USEARCH-UNOISE3 was also capable of single-base resolution, but was limited (due to the default setting of α parameter, see Eq 1, Methods) to single-base difference variants present at a no more than 8:1 abundance ratio. Thus, it was able to differentiate between the three *B*. *vulgatus* ASVs (ratio 5:1:1), but not between the two *C*. *beijenrickii* variants (ratio 13:1) (Fig 3). In effect, USEARCH-UNOISE3 (with default parameters) does not detect ASVs at 1 Hamming distance from another sequence that is >8 times more abundant.

USEARCH-UNOISE3 and Qiime2-Deblur were the only two pipelines to show perfect specificity on the mock sample sequencing data, producing no spurious OTUs/ASVs. Although showing the best sensitivity, DADA2 flows did produce some spurious ASVs. USEARCH-UPARSE and MOTHUR also produces some spurious OTUs. In MOTHUR, there was a visible effect of the cutoff applied to very low abundance sequences prior to clustering. While MOTHUR (DGC.0) and MOTHUR (DGC.1) both produced more spurious OTUs compared to USEARCH-UPARSE, the higher cutoff MOTHUR workflows (DGC.3 and Opticlust.3) produced fewer spurious features and were the OTU-level flows with the best specificity. In our analysis, all MOTHUR flows generated far fewer OTUs compared to QIIME-uclust, in contrast to a similar benchmark performed by the DADA2 authors [6]. It should be noted that USEARCH-UPARSE (by default) automatically removes singletons in the OTU clustering step.

Both DADA2 and USEARCH-UNOISE3 showed high accuracy in the quantification of abundance ratios in the case of 1-base-pair-difference ASVs (Table 2), yielding values close to the true ratios. Qiime2-Deblur did not differentiate any of the mock ASVs that were only 1 Hamming distance apart and thus did not demonstrate single-base resolution in this analysis.

**Table 2:** Inferred ratios of 16S rRNA gene variants. Expected ratios (based on known copy numbers of the respective 16S rRNA gene variants) are shown in bold. USEARCH-UNOISE3 could not differentiate the two *C*. *beijerinckii* variants. Qiime2-Deblur could not differentiate any of the variants.

	*B*. *vulgatus* variants			*C*. *beijenrijkii* variants
Expected ratio:	V1:V2 (5:1)	V1:V3 (5:1)	V2:V3 (1:1)	V1:V2 (13:1)
DADA2	5.60	5.23	0.94	14.28
USEARCH-UNOISE3	5.31	4.90	0.93	NA
Qiime2-Deblur	NA	NA	NA	NA

Extremely low abundance spurious OTUs/AVSs can be filtered out with relative ease in downstream analysis steps and may therefore have marginal impact on end results. However, QIIME-uclust flows assigned more than 12% of total counts to spurious OTUs, compared to at most 0.17% in other pipelines (Table 3). All three QIIME-uclust flows produced hundreds of spurious OTUs (around 25 times more than the number of true sequences in the mock sample), orders of magnitude more compared to other pipelines. While the flow using the external (and more stringent) quality control produced fewer spurious OTUs compared to the other two flows, the improvement was relatively small (around 10%).

**Table 3:** Proportion of counts assigned to either true or spurious OTUs/ASVs.

Pipeline	Counts in Exact ASVs/OTUs [%]	Counts in Spurious ASVs/OTUs [%]
QIIME-uclust	87.77	**12.21**
MOTHUR	99.83	0.17
USEARCH-UPARSE	99.84	0.16
DADA2	99.88	0.12
Qiime2-Deblur	100	none
USEARCH-UNOISE3	100	none

### Pipeline- and parameter-dependent biases

**Fig 4:**
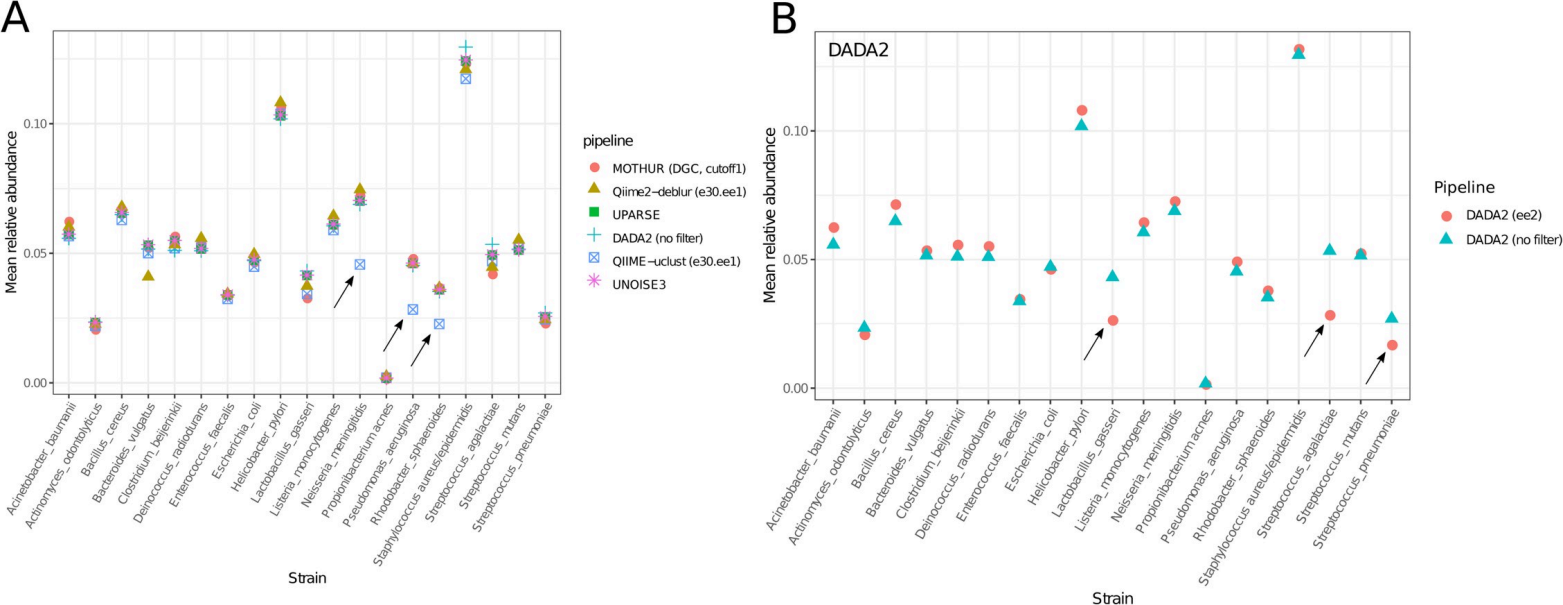
Inferred mock community composition. A) Comparison of QIIME-uclust vs. other pipelines. B) Comparison of DADA (no filter) vs. DADA2 (ee2). OTUs/ASVs whose abundance was under-estimated are indicated with arrows.

Biases affecting the inferred sample composition (systematic under- or over-estimation of certain taxa) pose a problem for amplicon sequencing bioinformatic pipelines, particularly if influenced by factors that can vary between samples or sequencing runs (e.g. read sequencing quality). We observed one such bias in the QIIME-uclust output (Fig 4A). While most workflows yielded very similar relative abundance values, all QIIME-uclust flows severely under-estimated the abundance of three OTUs (corresponding to *Neisseria meningitis*, *Pseudomonas aeruginosa*, and *Rhodobacter sphaeroides*). The bias was caused by QIIME-uclust assigning a large proportion of the counts of these true OTUs to other, spurious OTUs. This effect was independent of quality filtering parameters (i.e. it was observed in all three QIIME-uclust flows) and is likely intrinsic to the closed-reference OTU clustering specific to QIIME-uclust.

Another bias was induced in DADA2 (Fig 4B) by quality filtering. While the DADA2 (no filter) flow gave results in line with that of other pipelines (Fig 4A), the DADA2 (ee2) flow under-estimated the relative abundance of three ASVs (*Lactobacillus gasseri*, *Streptococcus agalactiae*, and *Streptococcus pneumoniae*). This bias was caused by preferential filtering (exclusion) of reads from these ASVs in the quality filtering step. While it is widely known that Illumina sequencing error rates are position-dependent (i.e. error rates tend to increase towards the end of the read), it is often neglected that they may also be affected by underlying sequence patterns [29]. Particular patterns of bases may result in much higher base call error rates than would be expected. Examples of such patterns are “GGC” triplets or inverted repeats (more than 8 bases long) located upstream of the respective position [29]. Thus, if a particular ASV sequence happens to contains such a pattern, application of a quality filter will exclude its reads preferentially before the denoising step. The V4 region of the 16S rRNA gene contains 8 instances of the “GGC” pattern for *L*. *gasseri*, 7 for *S*. *agalactiae*, and 9 for *S*. *pneumoniae*, though other patterns likely contribute to the effect. In practice, this presents an issue only for DADA2 (in the case of paired-end sequencing) since all other pipelines merge paired-end reads before clustering/denoising. In these other flows, the errors at the position where the pattern is present are corrected using information from the complementary read in the pair. Considering that the additional quality filter did not improve the specificity of the DADA2 pipeline (Table 2, Fig 2) while introducing a significant bias in the output (Fig 4B), we advise against it.

### HELIUS fecal sample dataset

#### Conversion of reads to counts

**Fig 5:**
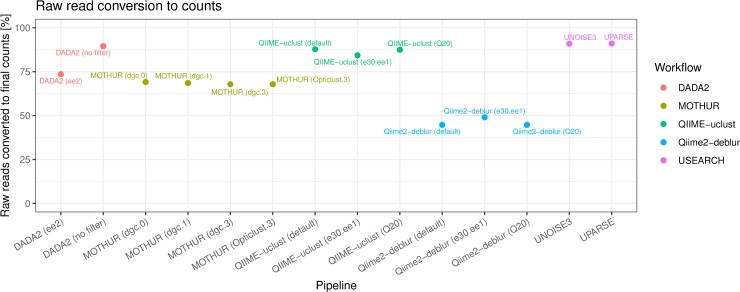
Raw reads conversion to final counts.

Large throughput is desirable to improve detection of low abundance taxa and to maximize the chance than samples with a lower number of sequencing reads will yield sufficient counts to be included in downstream analyses. In this study, we observed a tendency of Qiime2-Deblur to output far fewer counts than other pipelines (Fig 5). While other workflows converted more than 70% of reads form the mock community into counts (with highest conversion rate for USEARCH-UPARSE and USEARCH-UNOISE), Qiime2-Deblur flows converted less than 50%.

#### Quantitative comparison of pipeline outputs

**Fig 6:**
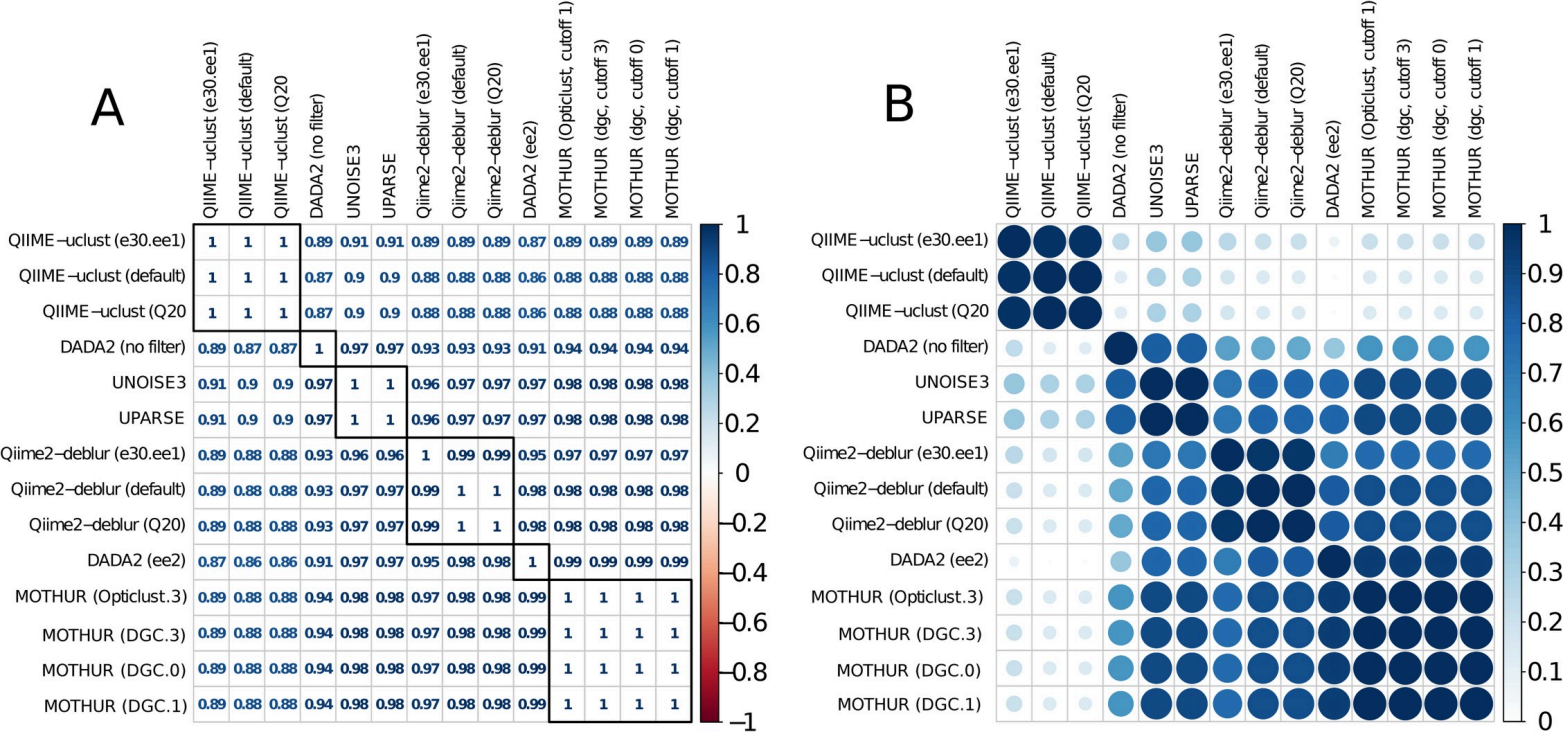
Spearman's rho correlation averaged across all samples of the HELIUS fecal sample dataset (N = 2170). A) Actual values. B) Values scaled to range between 0 and 1. Hierarchical clustering was applied to both rows and columns in order to group pipelines based on the degree of correlation of their outputs.

Agreement between the sample composition profiles produced by different pipeline flows was generally high (as measured by the median Spearman's ρ correlation across all OTUs) (Fig 6). For this comparison, DADA2, USEARCH-UNOISE3, and Qiime2-Deblur ASVs were clustered into 97% OTUs in order to be comparable to output from OTU-level pipelines. Different quality filtering parameters (tested in QIIME-uclust and Qiime2-Deblur) or clustering algorithm and cutoffs (tested in MOTHUR) had negligible effect on the inferred composition. The exception was DADA2, for which additional quality filtering shifted the composition profile. While different flows of the same pipeline were clearly grouped together when using hierarchical clustering (Fig 6), DADA2 (no filter) clustered next to USEARCH-UPARSE and USEARCH-UNOISE, while DADA2 (ee2) clustered together with the MOTHUR flows.

Table 4 shows read tracking for the different workflows as well as the total numbers of OTUs/ASVs produced from the HELIUS fecal sample dataset. Consistent with results from the mock community analysis, QIIME-uclust flows produced very large numbers of OTUs (around 200000). More stringent read quality filtering only reducing this number by approx. 25%. All QIIME-uclust flows produced an order of magnitude more OTUs compared to any other OTU-level workflow. Based on mock community results, the vast majority of these OTUs are expected to be spurious. USEARCH-UPARSE and both MOTHUR flows using cutoff 3 (Opticlust and DGC) produced a similar number of OTUs (ranging from around 4000 to 5500 OTUs) suggesting that this is the probable range for the number of true OTUs in this dataset. In contrast, QIIME-uclust produced between 150000 and 200000 OTUs. While the cutoff parameter had a large effect on the number or OTUs produced by MOTHUR, there was little to no effect of different quality filtering parameters on the number of ASVs produced by DADA2 and Qiime2-Deblur.

**Table 4:** Read tracking information and OTU/ASV outputs for the pipeline flows applied to the HELIUS data.

Pipeline Flow	Merged [% of raw]	Filtered [% of raw]	Clustered / Denoised [% of raw]	Conversion to OTUs/ASVs [% of filtered]	Conversion to OTUs/ASVs [% of raw]	Total no. of OTUs/ASVs	Non-singleton OTUs / ASVs	Singleton OTUs / ASV
**OTU-level**								
QIIME-uclust (default)	86.78	86.78	86.78	99.57	86.41	201735	201735	0
QIIME-uclust (Q20)	86.78	86.41	86.41	99.66	86.12	195377	195377	0
QIIME-uclust (e30.ee1)	93.01	82.90	82.90	99.91	82.82	150752	150752	0
MOTHUR (DGC.0)		70.48	66.37	94.17	66.37	23347	16161	7186
MOTHUR (DGC.1)		70.48	66.39	94.19	66.39	12822	12640	182
MOTHUR (DGC.3)		70.48	65.47	92.89	65.47	4022	3832	190
MOTHUR (Opticlust.3)		70.48	65.47	92.89	65.47	5302	5053	249
USEARCH-UPARSE	93.01	82.90	82.90	96.60	89.85	5559	5557	0
**ASV-level**								
DADA2 (ee2)		73.82	71.26	95.00	70.13	26763	26763	0
DADA2 (no filter)		98.12	90.11	90.45	88.75	24469	24469	0
Qiime2-Deblur (default)	74.53	74.53	74.53	51.50	38.39	11120	11120	0
Qiime2-Deblur (Q20)	74.53	74.53	74.53	51.50	38.38	11120	11120	0
Qiime2-Deblur (e30.ee1)	93.01	82.90	82.90	51.04	42.31	11735	11735	0
USEARCH-UNOISE3	93.01	82.90	82.90	97.48	90.67	7659	7519	140

Qiime2-Deblur produced far fewer counts than other pipelines (Fig 5, Table 4), while QIIME-uclust, USEARCH-UPARSE, and USEARCH-UNOISE3 had conversion rates of more than 90% of initial raw reads. The low conversion rate of Qiime2-Deblur flows is due to the “count substraction”-based algorithm of Deblur [7], which removes more than 50% of the (filtered reads) counts entering the denoising step (Table 4). The proportion of chimeric reads removed by the different pipelines was very similar, averaging around 1% of raw read counts.

**Fig 7:**
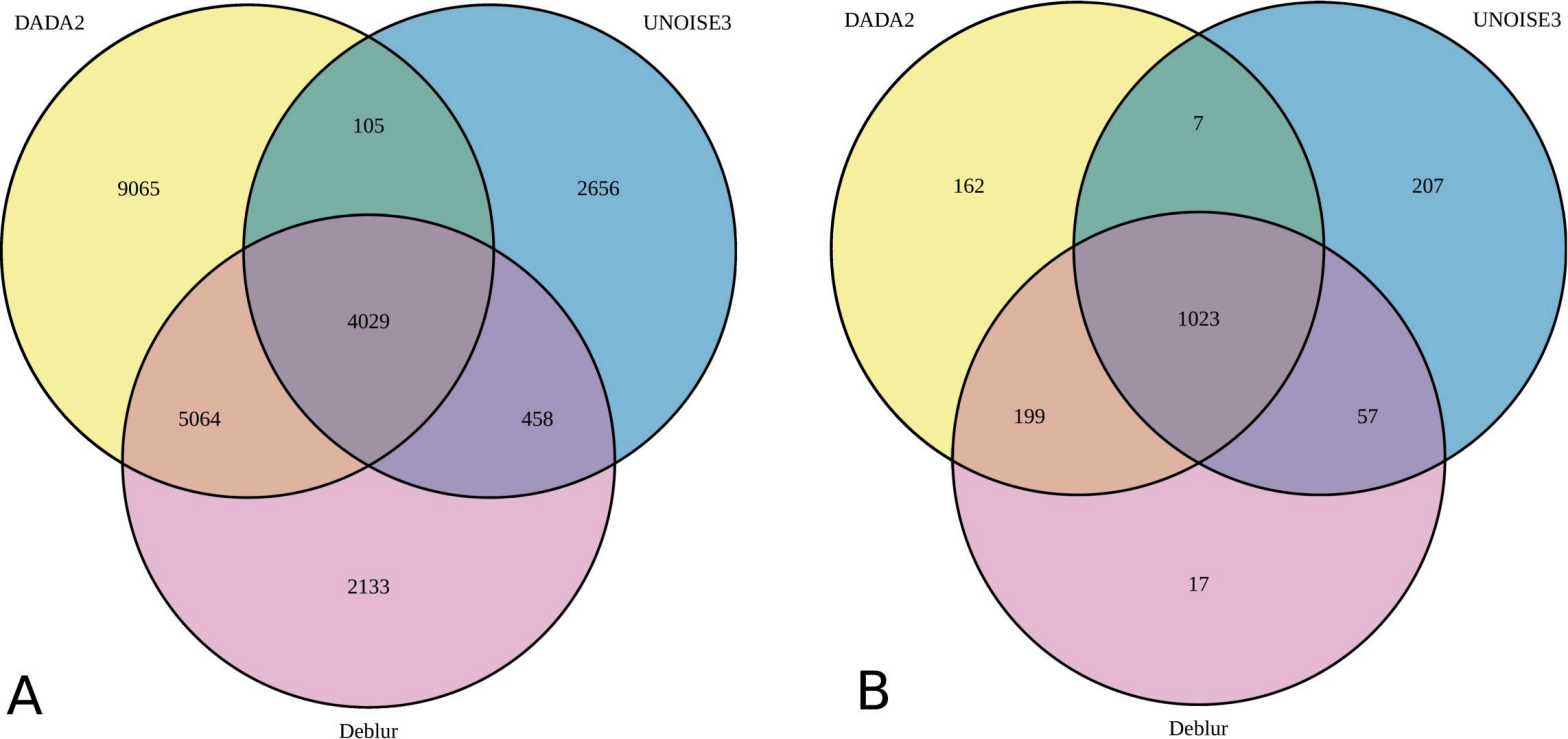
Venn diagram showing the overlap between the ASVs produced by three denoising pipelines from the HELIUS fecal sample data (N = 2170). **Workflows shown are DADA2 (no filter), Qiime2-Deblur (e30.ee1), and USEARCH-UNOISE3**. A) ASVs remaining after rarefaction to 10 000 counts. B) Filtered ASVs (mean relative abundance of at least 0.002% of rarefied counts).

In the HELIUS fecal sample dataset analysis there was a 3.5-fold difference between the highest number of ASVs produced by a pipeline (around 25000, in DADA2) and the lowest number (more than 7500, in USEARCH-UNOISE3). Qiime2-Deblur produced around 11000 ASVs (Table 4). The representative sequence of an OTU may vary depending on the nature of the (*de novo*) clustering algorithm and is influenced by other sequences present, particularly in complex samples. However, by definition, ASV sequences are exact representations of biological sequences and are therefore directly comparable between workflows. We compared ASV sequences from each of the three ASV-level pipelines, identified perfect matches, and constructed Venn diagrams showing the overlap between the different outputs (Fig 7). Only around 4000 ASVs were found by all three pipelines (Fig 7A). Around 9000 (around 37% of total) ASVs produced by DADA2 were not found by either USEARCH-UNOISE3 or by Qiime2-Deblur. This mirrors findings from the mock community analysis, where DADA2 showed both the best sensitivity and the highest propensity for spurious ASVs among the three ASV-level pipelines. USEARCH-UNOISE3 and Qiime2-Deblur ach produced more than 2000 ASVs not found by other pipelines. These differences were mostly associated with low-abundance ASVs. To illustrate, 95.5% of counts produced by DADA2 were assigned to ASVs found by USEARCH-UNOISE3, while 98% of counts produced by USEARCH-UNOISE3 were assigned to ASVs found by DADA2. ASVs shared by USEARCH-UNOISE3 and Qiime2-Deblur accounted for around 98% of total counts. Thus, while the number of non-consensus ASVs produced by the ASV-level pipelines was large, it accounted for only 2% to 4% of the total number of counts.

The creation of spurious OTUs/ASVs is a known issue for 16S rRNA bioinformatic pipelines. Some authors recommend applying a minimum relative abundance threshold filter in order to remove OTUs/ASVs with extremely low abundance that have a higher probability of being spurious. When a 0.002% minimum relative abundance filter was applied to the ASVs tables, most non-consensus ASVs were removed (Fig 7B). From each pipeline, 1396 ASVs (DADA2), 1294 ASVs (UNOISE3), and 1296 ASVs (Qiime2-Deblur) passed the filter, of which 1023 ASVs were found by all three pipelines. Thus, around 26% of filtered DADA ASVs, 21% of filtered UNOISE3 ASVs, and 21% of filtered Qiime2-Deblur ASVs were found by at most two of the three pipelines (i.e. were non-consensus ASVs). An analysis of closest matches between the (filtered) ASVs produced by the different pipelines showed that while DADA2 and Qiime2-Deblur non-consensus ASVs were generally 1 base away from the closest UNOISE3 ASVs, the most common closest match for UNOISE3 non-consensus ASVs was at 3 or 4 bases distance (S1 Fig). Thus, pipeline-specific biases remained after the application of a typical low-abundance filter. Moreover, the filter also removed around 75% of the 4029 consensus ASVs. While abundance-based filters may remove some of the spurious ASVs, they will also remove many true low-abundance biological features.

**Fig 8:**
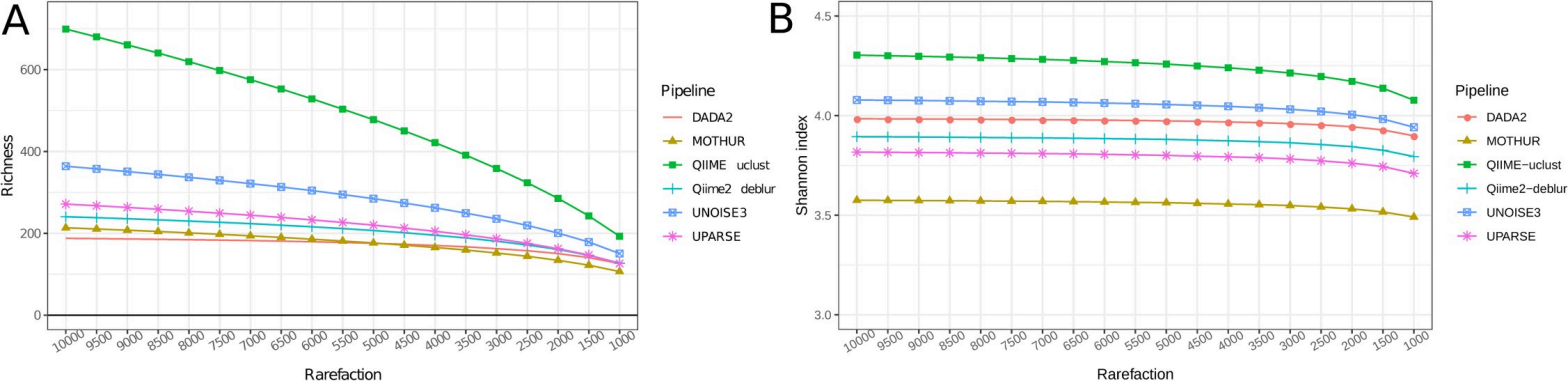
Alpha-diversity measures at different rarefaction levels. Values shown are averages across all samples in the HELIUS fecal sample dataset. A) Sample richness (no. of OTUs/ASVs per individual sample). B) Shannon index. Only one workflow from each pipeline is shown: DADA2 (no filter), QIIME-uclust (e30.ee1), Qiime2-Deblur (e30.ee1) and MOTHUR (DGC.1).

**Fig 9:**
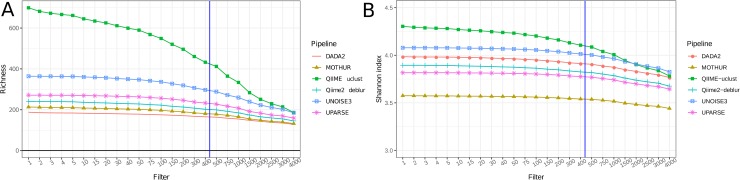
Alpha-diversity measures after downstream filtering of very low-abundance OTUs/ASVs. X-axis shows the no. of counts that an OTU/ASV must reach (in the entire dataset) in order to be retained. All OTU/ASV tables rarefied to 10000 counts / sample prior to filtering. Values shown are averaged across all samples in the HELIUS fecal sample dataset. A) Sample richness. B) Shannon index. The blue vertical bar marks the filter threshold corresponding to 0.002% of rarefied counts.

There was a significant effect of the pipeline on downstream alpha-diversity measures (Fig 8). Ground-truth ASV-level data should always yield higher alpha-diversity than OTU-level data. However, two types of errors can bias perceived alpha-diversity. First, as observed in QIIME-uclust workflows, massive numbers of spurious OTUs can greatly inflate perceived alpha-diversity. Spurious OTUs are responsible for QIIME-uclust yielding much higher alpha-diversity values than all others pipelines, including all ASV-level workflows. The relatively steep downward slope observed for QIIME-uclust when plotting richness as function of rarefaction level or of abundance-based OTU filtering (Figs 8A and 9A) is indicative of large numbers of very low-abundance OTUs, most of which are likely spurious (Figs 2 and 3). The propensity of QIIME-uclust to generate spurious OTUs and inflate alpha-diversity measures has been previously reported by other authors [6, 30]. Second, as observed for QIIME2-Deblur, an ASV-level pipeline can fail to distinguish very closely related true biological sequences and clump them together into a single ASV. This will artificially decrease perceived alpha-diversity compared to higher sensitivity ASV-level pipelines, and is the reason why Qiime2-Deblur yielded lower alpha-diversity values compared to DADA2 and USEARCH-UNOISE3 (Fig 8B). Pipeline-induced biases (e.g. inflation of sample richness and diversity) cannot be fully addressed using filters (Fig 9). We applied a wide range of filters to the OTU/ASV tables and observed that inter-pipeline differences in alpha-diversity measures remain after the application of typical filters (i.e. 0.002% to 0.005% [31] of relative abundance).

## Conclusion

Large differences in sensitivity and specificity were observed between different pipelines. DADA2 showed the best sensitivity and resolution (followed by USEARCH-UNOISE3) at the cost of producing higher number of spurious ASVs compared to USEARCH-UNOISE3 and Qiime2-Deblur. USEARCH-UPARSE and MOTHUR produced similar numbers of OTUs, especially when a cutoff value was used in MOTHUR to remove singletons or extremely low abundance sequences before clustering. QIIME-uclust workflows produced huge numbers of spurious OTUs as well as inflated alpha-diversity measures, regardless of quality filtering parameters. Current QIIME users may consider switching to other pipelines. Indeed, the authors of QIIME have stopped supporting the platform since 1st January 2018 and are encouraging users to switch over to Qiime2. Biological conclusions based on alpha-diversity measures obtained from QIIME-uclust pipelines may warrant revisiting or confirmation other pipelines. ASV-level workflows offer superior resolution compared to OTU-level, and in this study showed better specificity and lower spurious sequence rates. Moreover, ASV-level pipelines allow for easier inter-study integration of biological features, as ASVs have intrinsic biological meaning, independent of reference database or study context [9].

We found DADA2 to be the best choice for studies requiring the highest possible biological resolution (e.g. studies focused on differentiating closely related strains). However, USEARCH-UNOISE3 showed arguably the best overall performance, combining high sensitivity with excellent specificity.

Current advances in sequencing technology and bioinformatic pipelines offer new opportunities for ecologists, microbiologists and biomedical scientists. This paper aimed to guide researchers in their choice of the pipeline most suited for their goal while pointing out some of the associated pitfalls and limitations.

## Supporting information

S1 Fig Levenshtein distance from the the ASVs of each pipeline (DADA2, USEARCH-UNOISE3, and Qiime2-Deblur) to the closest ASV in another pipeline's ASV output. Data is shown for the rarefied ASV tables, filtered using a minimum relative abundance threshold (0.002%). For the Levenshtein distance calculation, DADA2 and UNOISE3 ASVs were trimmed to 250 bp to match the length of Qiime2-Deblur ASVs (which are trimmed to 250 bp in the pipeline flow).
(TIF)

S1 Table Description of mock sample composition. Accession number, 16S rRNA gene copy number, and number of 16S rRNA gene amplicon variants are given for each mock community member.
(XLSX)
