# Supplementary figures and images for: Comparing bioinformatic pipelines for microbial 16S rRNA amplicon sequencing
Source: PLoS One. 2020 Jan 16;15(1):e0227434. doi: 10.1371/journal.pone.0227434 (PMC6964864; doi:10.1371/journal.pone.0227434)

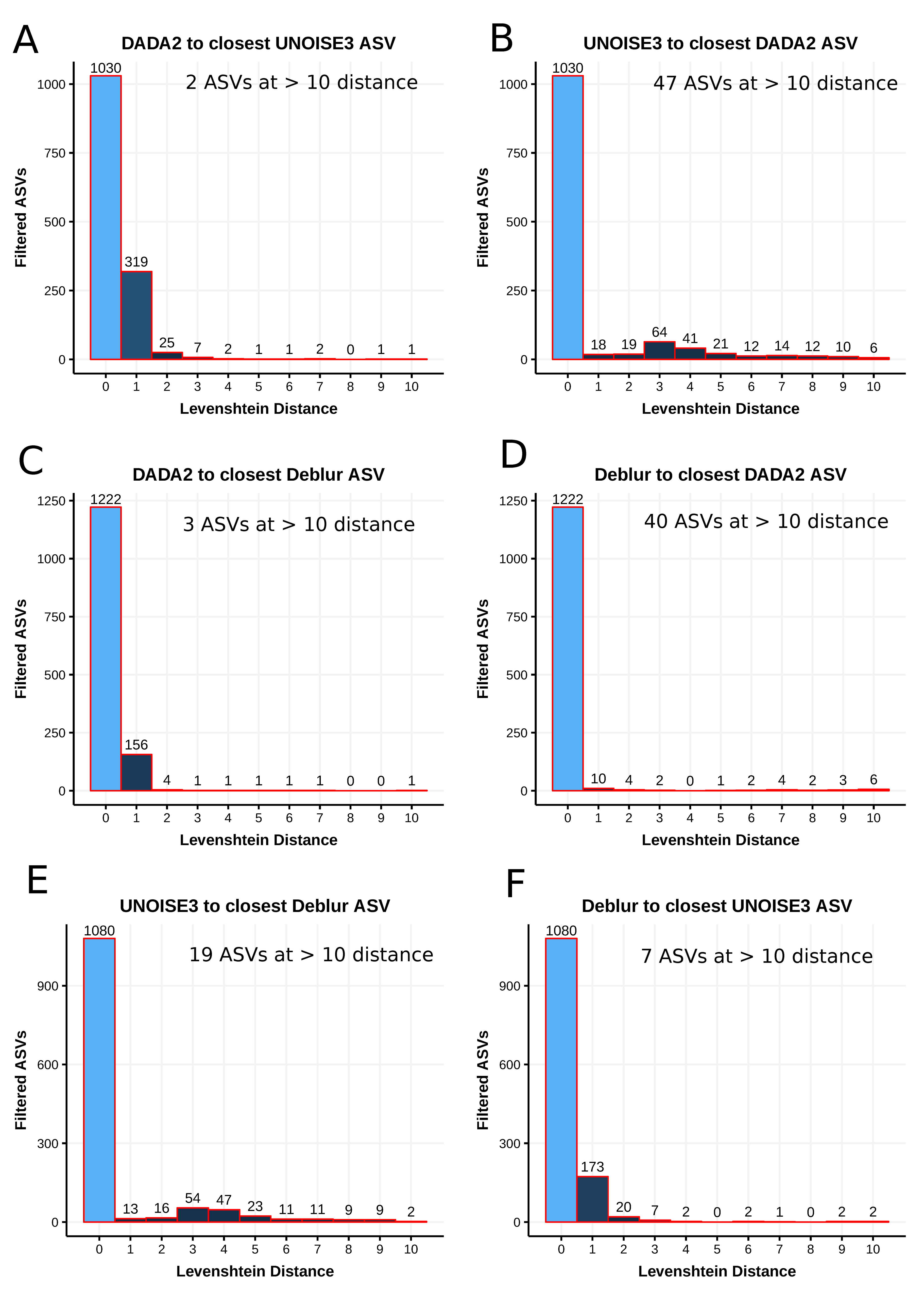

Supplement: S1 Fig — Data is shown for the rarefied ASV tables, filtered using a minimum relative abundance threshold (0.002%). For the Levenshtein distance calculation, DADA2 and UNOISE3 ASVs were trimmed to 250 bp to match the length of Qiime2-Deblur ASVs (which are trimmed to 250 bp in the pipeline flow). (TIF) [file pone.0227434.s001.tif]
